# pH-Dependent Non-Covalent Release of Chemotherapy from Carriers

**DOI:** 10.24976/Discov.Med.202436182.42

**Published:** 2024-03

**Authors:** Qixin Leng, Aishwarya Anand, Archibald James Mixson

**Affiliations:** 1 Department of Pathology, University Maryland School of Medicine, University of Maryland, Baltimore, MD 21201, USA

**Keywords:** tumor, pH, carriers, non-covalent, chemotherapy, doxorubicin, methotrexate

## Abstract

Although Warburg discovered pH discrepancies between tumor and normal tissues nearly 100 years ago, developing therapies to take advantage of this concept was relatively slow for the first 70 years. During the last 30 years, there has been an exponential increase in the use of pH-dependent strategies for both low molecular weight drugs and nanoparticles. Two frequently discussed approaches are the chemotherapy’s release from pH-sensitive covalent linkages of macromolecules or from pH-dependent disruption of charged polymeric nanoparticles. In contrast, pH-dependent non-covalent bonds between the chemotherapy agent and macromolecules have rarely been discussed, yet this underappreciated strategy has great potential. These non-covalent interactions are primarily ionic or hydrogen bonds with supporting roles from hydrophobic bonds. In addition to the facile coupling of the drug with the carrier, these non-covalent interactions may show marked pH dependence. Consistent with pH dependence, many of these drug-loaded carriers showed significant *in vitro* and, in some cases, striking *in vivo* activity. In this review, we will focus on pH-sensitive non-covalent bonds, highlighting the release of drugs from diverse carriers such as tetrahedron DNA structures, cyclodextrin, polymeric carriers, and carbon-based quantum particles.

## Introduction

A promising approach to augment cancer therapeutics is based on the low extracellular pH of solid tumors. The nearly universal low extracellular pH (pHe) of tumors was initially thought to be due to increased glycolysis of tumor cells [1,2]. Several other likely mechanisms of the low pHe include an increase in membrane proton pumps, bicarbonate transport exchanges, sodium-hydrogen exchanges, and carbonic anhydrase activity [3-6]. Our understanding of these mechanisms continues to evolve. Moreover, the pHe varies between non-necrotic tumors and is likely due to several interdependent factors, including the cells’ proximity to the vasculature, the tumor size, the buffering capacity of the tumor, the degree of hypoxia, and the heterogeneity of the glycolytic activity within the tumor. The pHe of solid tumors ranges from 6.4 to 7.3 with an average of 6.8 [7,8].

Previous studies have noted that the tumor’s extracellular pH may influence the efficacy of cancer chemotherapeutic agents [9,10]. For example, by lowering the pH of the cancer cell media, the antitumor effectiveness of camptothecin and its analogs improved by stabilizing the lactone ring [9]. Alternatively, raising the tumor’s extracellular pH increases the antitumor efficacy of doxorubicin (Dox) by decreasing its charge and improving its ability to cross the cellular membrane [10]. Based on these studies with low molecular weight chemotherapeutic agents, pH-dependent nanoparticles have been developed to deliver cancer chemotherapeutic agents [11].

Although the lower pHe of the tumor may enhance the efficacy and specificity of the chemotherapy, its mild acidity may not be sufficient to disrupt the nanoparticle or release the drag [12,13]. Compared to the pHe of tumors, endosomes become significantly more acidic and reach a pH between 5 and 6.5 [13]. As a result, the lower endosomal pH has a key role in augmenting the release of chemotherapeutic drugs from nanoparticles [14].

There are two primary pH approaches targeting tumors with chemotherapy-loaded macromolecules and nanoparticles (see reviews by Deirram *el al.* [11] and Leng *el al.* [13]). These approaches are often combined. One strategy is the protonation and repulsion of polymeric units of the nanoparticle in acidic pHs, disrupting the particle and releasing the drug. Second, a pH-sensitive covalent linkage between the drug and macromolecule is also a common tumor-targeting method. The pH-sensitive covalent bonds include imines, hydrazone, oximes, ketals, and orthoesters linkages, which break under acidic conditions [15]. These pH-dependent approaches may release a single drug [16] or a combination of drugs that act in concert against the tumor [17,18]. Some of these pH-sensitive chemical linkages or disassembly of NPs break apart at pHs between 6.0 and 7.0, consistent with the extracellular pH of the tumor [11,19,20]. More frequently, NP disassembly and covalent bonds are disrupted at more acidic pHs between pH 5.0 and 5.5, compatible with the pH of late endosomes. Notably, covalent bonds have a range of lability that extends beyond the optimal pH of lability. Hydrophobic environments may stabilize these pH-dependent covalent bonds and reduce the premature release of the drug from the macromolecule [21]. Still these non-covalent hydrophobic interactions serve a supporting role for pH-sensitive covalent bonds.

**Fig. 1.**
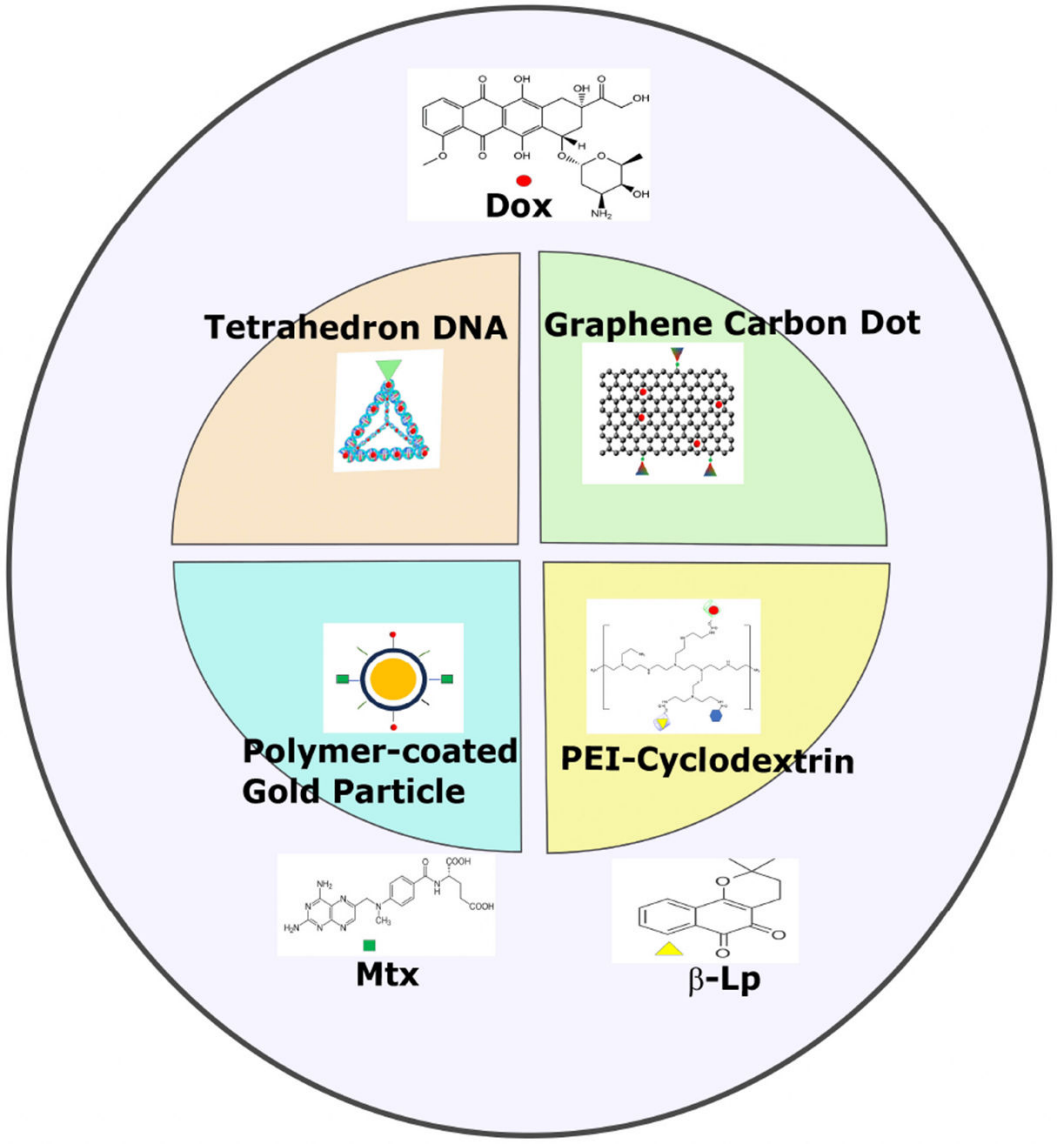
Schematic overview of carriers of drugs in this review. Several representative carriers (tetrahedron DNA, Polyethyleneimine (PEI)-Cyclodextrin, polymer-coated gold particles, and graphene carbon dots) demonstrated pH release of drugs, including doxorubicin (Dox), methotrexate (Mtx), or *β*-lapachone. The figure was created using Inkscape Version 1.2 (Inkscape Project, Boston, MA, USA) and ChemDraw Version 22.2 (Perkin Elmer, Waltham, MA, USA).

This review will focus on the release of the therapeutic agent from the carrier by disrupting the pH-sensitive non-covalent bonds. These pH-dependent non-covalent interactions, based primarily on ionic and hydrogen bonds [22], have received minimal attention, yet early studies have shown promise. While Dox has been the most frequent drug released by the carrier by these pH-dependent non-covalent interactions, other chemotherapeutic drugs such as *β*-lapachone (*β*-Lp) and methotrexate (Mtx) showed pH-dependent release [23-25]. This review will discuss four diverse carriers, DNA, cyclodextrin, polymeric nanoparticles, and carbon-based dots, that exemplify these pH-sensitive non-covalent drug releases [24-27] (Fig. 1).

## Carriers

### DNA Structures: Micelles, Tetrahedrons, and Rolling Circles

DNA was one of the earliest carriers of anthracyclines to demonstrate antitumor activity [28,29]. Most articles indicate that Dox binds with higher affinity to adjacent GC base pairs (bp) [30-32]. but despite the high affinity, the bound Dox is in equilibrium with free Dox [33,34]. Because the pKa of the phosphodiester is between 0–1 and the pKa of nucleobases is not between 5 and 8, few studies have examined whether the binding between nucleic acids was pH-sensitive [35,36]. Nevertheless, some DNA structures and formulations have demonstrated enhanced release of the intercalated Dox at acidic pHs.

Only one of the pH-sensitive DNA carriers of chemotherapy has been extended from *in vitro* inhibitory to *in vivo* tumor studies. Charbgoo *et al.* [23] incorporated Dox via intercalation inside targeted “DNA micelles” to inhibit cancer cells. A single-strand oligodeoxynucleotide-cholesterol conjugate and a complementary cDNA strand-KLA peptide conjugate formed the micelles. Furthermore, these micelles targeted the Muc1 receptor, which is overexpressed in several cancers. These micelles in which Dox was intercalated into the DNA and the pro-apoptotic peptide, the D-form of KLA ((KLAKLAK)_2_), enhanced the cytotoxicity toward MCF-7 cancer cells compared to free Dox or KLA-only micelles. With one dose (2.5 mg/kg) administered intravenously, these micelles inhibited C26 tumor growth and prolonged the survival in mice more than the free Dox treatment group (*p* < 0.05). Specifically, the targeted micelle prolonged survival, with 60% of mice alive at day 30, whereas all mice in the Dox-alone treated groups had died. There was a significant difference in pH-sensitivity of Dox release from DNA micelles, particularly during the first 2 hours between pH 5.5 and 7.4. While 35% of Dox was released at pH 5.5, about 15% was released at 7.4 [23].

**Fig. 2.**
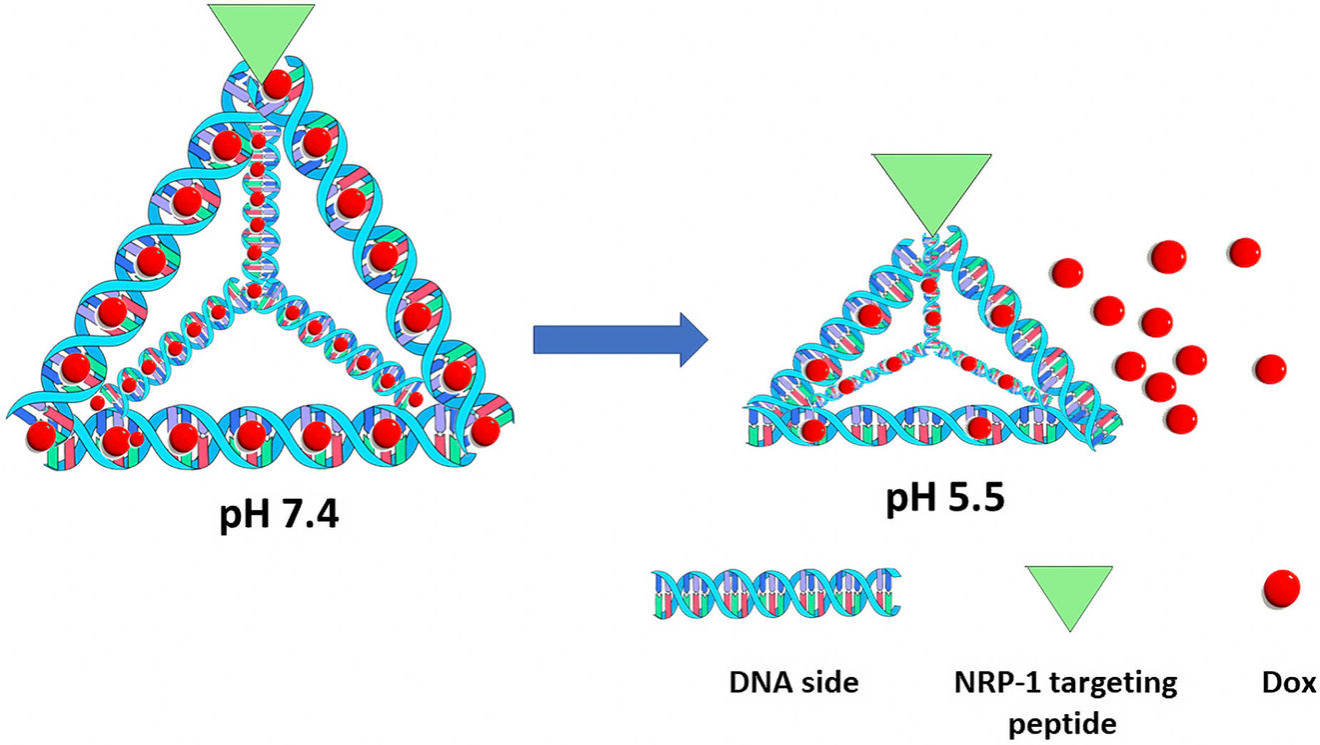
Dox-loaded DNA tetrahedron structure with release of Dox at an acidic pH. The figure was created using Inkscape Version 1.2 (Inkscape Project Boston, MA, USA).

In addition to the micelle comprised of complementary oligonucleotides, a small 15-nm tetrahedron-shaped DNA carrier that incorporated Dox also showed pH dependency [24] (Fig. 2). A tumor-penetrating peptide targeting neuropilin-1 (NRP-1) was conjugated to the tetrahedron. Targeting NRP-1, which is overexpressed in tumor endothelial cells and tumor cells, offers the possibility of transporting the Dox-loaded particle efficiently and deep into the tumor. Approximately forty and forty-five Dox molecules were in the targeted and untargeted tetrahedron, respectively. In contrast, a linear double-stranded 55-base pair DNA control incorporated about 13 Dox molecules, indicating that the tetrahedron structure efficiently loaded Dox.

Moreover, the tetrahedron structure was relatively stable to biological media compared to degradation of double-stranded DNA structure within 4 hours. Consistent with its higher uptake than the untargeted tetrahedron, the targeted structure had a greater inhibitory effect on glioblastoma cells (over a wide range of Dox concentrations). The IC50 for targeted and untargeted Dox-loaded tetrahedrons were 2.48 and 4.78 μM, respectively. At pH 5, which approximates the pH of late endosomes, nearly 50% of the Dox was released at 24 h from the tetrahedron, whereas at pH 7.4, about 25% was released. Because the tetrahedron ultimately localizes to the lysosomes, further Dox release would be expected upon enzymatic digestion of DNA. Similar pH release patterns of Dox were observed with the low molecular weight DNA control. The authors speculated that the pH-dependent release of Dox was due to structural changes in the tetrahedron DNA and DNA control. Indeed, the authors previously determined that the low molecular weight tetrahedron and DNA showed shrinkage and stretching at different pHs [36]. A second rationale for the greater release of Dox from the tetrahedron structure (and other carriers in this review) was its higher aqueous solubility at lower pHs [24].

One potential obstacle is that the released Dox, with a pKa of 8.3, will become progressively protonated and hydrophilic as the Dox-loaded tetrahedron traverses the endocytic pathway to the lysosomes [24,37]. Inside more acidic vesicles, the highly charged Dox will have decreased passive membrane transport compared to less acidic vesicles. Slight modifications in the extracellular pH have previously been demonstrated to significantly affect the passive transport and cellular toxicity of Dox [4,32]. Thus, Dox released in less acidic vehicles (i.e., pH 6.5–7.0) may enhance its accumulation in the cytosol. Alternatively, membrane-disrupting NPs could enable the escape of Dox into the cytosol independent of the protonation of Dox [13]. As currently designed, the tetrahedron structure and other carriers (i.e., carbon dots) do not have a membrane-disrupting function.

The two previously mentioned Dox-loaded DNA examples demonstrated pH sensitivity. The common thread shared by these pH-sensitive structures was that they have relatively low molecular weight. In addition to the tetrahedron structure [24], Charbgoo *el al.* [23] showed pH sensitivity drug release from low molecular weight oligodeoxynucleotide nanoparticles. Thus, the non-covalent pH-dependent mechanism of stretch and shrinkage with the release of Dox from the low molecular weight DNA structures seemed the likely explanation [36]. Somewhat supporting this idea, we did not discover pH-sensitivity for Dox release from the pseudo-random adjoining GC sequences in large molecular weight plasmids [38].

Under special circumstances, however, Dox has exhibited pH-dependent release from large molecular weight DNA structures. For example, Zhang *el al.* [39] intercalated Dox in a rolling circle DNA to target prostate membrane specific antigen (PMSA)-expressing prostate cancer cells. The rolling circle incorporated repeating low molecular weight motifs, containing a PMSA aptamer, a pH-sensitive AT-rich spacer to release the Dox, and a Dox-intercalated GC-enriched segment. The loading capacity and encapsulation efficiency of Dox in the particle was 34.7% and 60.3%, respectively. Moreover, the targeted NPs were taken up by PMSA-positive cells significantly more than PMSA-negative cells. The rolling circle NP had a mean size of 234 nm and released Dox readily in an acidic environment compared to a physiological environment. Specifically, 80% of the Dox was released by 210 min at pH 5.4, while 20% was released at pH 7.4. The AT-rich domain facilitated Dox release from the GC segment [40]. Without the AT-rich segment juxtaposed to the GC-rich domain, the rolling circle did not show a pH-dependent release of Dox [40,41].

Despite the pH sensitivity release of Dox from DNA demonstrated by these studies directly or indirectly, some may only have clinical utility if the binding of Dox with DNA is further stabilized. For example, during 24 hours at neutral pH, Dox release from micelles and the tetrahedron structures was 20 and 25%, respectively. Further stabilization of these interactions may be accomplished with pH-sensitive acetal bonds [40,42].

### Cyclodextrin

**Fig. 3.**
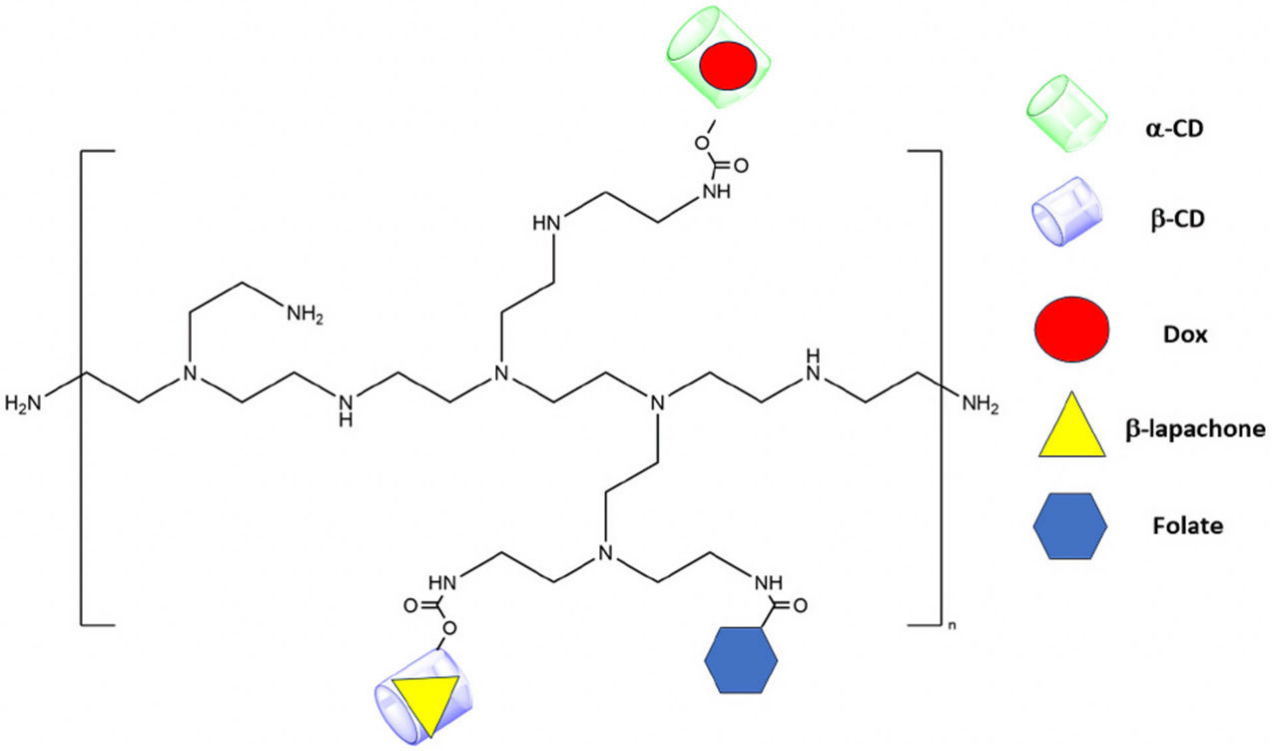
Folate ligand PEI conjugated to cyclodextrins. Dox and *β*-lapachone bind to the doughnut-shaped hydrophobic cavities of *α* and *β* cyclodextrins. The size of the drug-loaded PEI-cyclodextrin structure was 367 nm. The figure was created using ChemDraw Version 22.2 (Perkin Elmer, Waltham, MA, USA).

In contrast to DNA, in which interactions with Dox were base-specific, cyclodextrin has structural cavities to bind with drugs. Kowalczyk *el al.* [25] loaded two cancer chemotherapy hydrophobic drugs, Dox and *β*-lapachone, into the doughnut-shaped hydrophobic cavities of *α* and *β* cyclodextrins, respectively. *β*-lapachone is a quinone-containing compound that has shown promise against various cancers by inhibiting their topoisomerase I activity. Whereas Dox binds primarily to the a-cyclodextrin cavity, the *β*-lapachone binds to the *β*-cyclodextrin cavity. The cyclodextrins were covalently linked to a folate ligand-Polyethyleneimine (PEI) conjugate (Fig. 3).

Interestingly, both drugs were retained within the cyclodextrin cages for 15 hours at pH 7.4 (less than 2.5% release). In contrast, about 20–25% and 50–55% of the drugs were released from the cages at pH 5.5 and 4.0, respectively. Quartz crystal microbalance with dissipation monitoring (QCM-D) studies suggested that the lower pH degraded the cyclodextrin carrier, releasing the drugs. However, other studies have indicated that cyclodextrins were stable in mild acidic environments. The instability of cyclodextrins was primarily due to ring opening and occurred at a very low pH and high temperatures (t_1/2_~15 hours for ring opening at pH 1.1 at 70 °C) (see review by Loftsson and Brewster [43]). Consequently, the mechanism of how pH affects the release of these two hydrophobic drugs from the cyclodextrin is not evident to us. Notably, pH does affect the binding of some drugs to cyclodextrins [43].

One attractive feature of this conjugate was that PEI became progressively protonated in more acidic environments, providing the potential of the charged PEI to disrupt the acidic vesicle membranes. The folate-PEI-cyclodextrin conjugate carrier of Dox and *β*-lapachone had inhibitory activity (IC_50_) *in vitro* about 10-fold less than either free drug alone. Thus, with minimal to moderate release of the drugs at mildly acidic pHs, modifications of the cyclodextrin carrier will be required to augment their release.

### Polymeric Nanoparticles

As stated previously, chemotherapy release from polymeric nanoparticles is frequently due to the charge repulsion of the polymers. Consequently, the polymers become progressively protonated at acidic pHs, leading to nanoparticle instability and chemotherapy release. In contrast, some polymeric nanoparticles release chemotherapy based directly on their pH-dependent non-covalent interactions [26,44,45].

For instance, Cunningham *el al.* [44] examined hydrophobic and ionic interactions between Dox and the carrier. They prepared three Pegylated micelle formulations varying in size between 30 and 60 nm, which were comprised of star-shaped cholic acid-based block copolymers. One of these micelles, further modified with carboxyl groups, showed marked pH dependency release of Dox. At an acidic pH of 5, the carboxyl groups of the nanoparticle became protonated with the release of nearly 90% of the Dox (by 12 hours). Furthermore, the particle was quite stable, with only 13% release after three days. Despite the significant pH dependency, these Dox-loaded micelles (11.4 μM) had an IC_50_ similar to that of the neutral hydrophobic, relatively pH-independent micelles (13 μM) for HeLa cells. Both formulations were significantly less effective in reducing cells’ viability than free Dox (IC_50_-2.2 μM). The utility of these different formulations will only be possible to evaluate with further *in vivo* efficacy and toxicity studies.

**Fig. 4.**
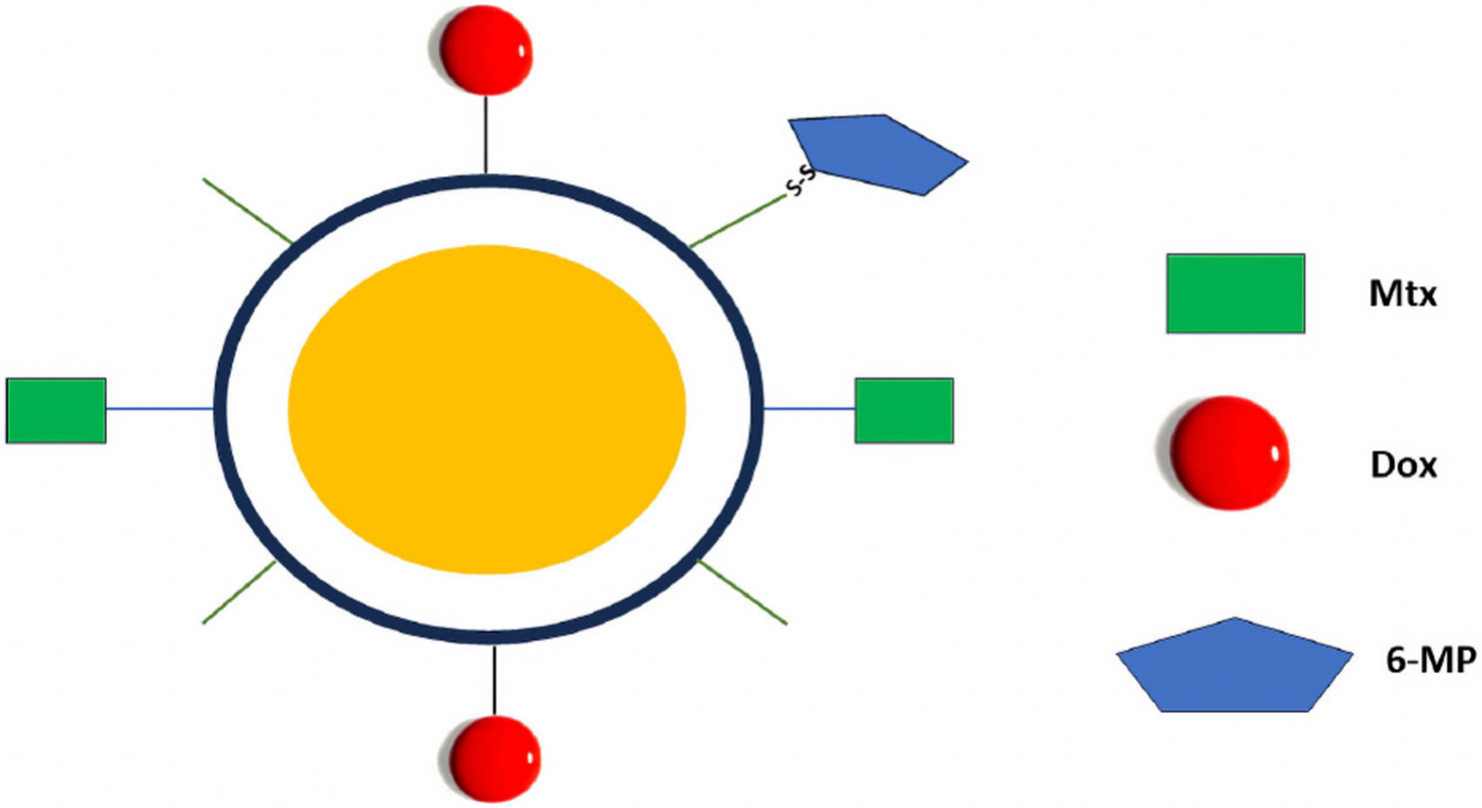
Drug-loaded polymer-coated gold particle. While the release of Mtx and Dox was pH-dependent, the release of 6-mercaptopurine (6-MP) was redox-dependent. The figure was created using Inkscape Version 1.2 (Inkscape Project, Boston, MA, USA).

Ghorbania and Hamishehkar developed an interesting, PEGylated polymer-coated gold particle that incorporated three drugs: Dox, methotrexate (Mtx), and 6-mercaptopurine (6-MP) [26]. In contrast to the release of redox-dependent 6-mercaptopurine, both Dox and Mtx were ionically bound to the polymer, and their release was pH-dependent (Fig. 4). At neutral pH, the ionized carboxyl groups of the coating polymer interacted with Dox, while the charged amino groups (of the polymer) interacted with the negatively charged Mtx. During a 24-hour period, there was approximately a 22% and 44% release difference between pH 7.4 and 5.0 in Dox and Mtx, respectively. Moreover, the 78 nm polymeric-coated gold particle loaded with the three drugs showed synergistic inhibition against several cancer cell lines. If this polymer-coated particle demonstrates *in vivo* efficacy, the stability and scalability of these nanoparticles with multiple components must be established for clinical trials.

### Carbon-Based Dot Delivery Systems

Discovered fortuitously in 2004 [46], carbon dots (CDs) are spherical nanocarriers less than 10 nm in size with low toxicity [47]. Carbon dots are synthesized by either a top-down approach, which involves the breakdown of large carbon nanostructures, or a bottom-up approach that utilizes small organic molecules [48-51]. Due to their fluorescence, high water solubility, and accessible surface modification, carbon dots have been increasingly used as a theranostic agent to determine biodistribution and to carry genes and chemotherapy drugs [49-52]. Although most carbon-based nanoparticle surfaces require modification with polymers to release the chemotherapy agent in acidic environments [53], we will discuss a notable exception with a potential advantage. If carbon dots do not require the addition of coating polymers to affect their pH release properties [53], they will remain small, enabling greater tumor penetration.

Hailing *el al.* [52] prepared carbon dots by microwave carbonization of the PEI polymer and glycerol. The size of these carbon dots varied between 2 and 8 nm, but the size of Dox-loaded CD was not stated in the report. Interestingly, the surface charge of the PEI-carbon dots was negative, indicating a large number of carboxyl groups on the surface. While the release of Dox from the PEI-CD was negligible at neutral pH, its release at pH 5.0 was significantly higher. This was likely due to the protonation of the carboxyl group and enhanced solubility of Dox. The increased protonation of carboxyl groups likely reduces the ionic interaction with the charged amino group and polar hydrogens of Dox. Alternatively, other studies have suggested that protonation of PEI or fragments of PEI on the surface of the CD might result in the release of the Dox [54]. However, no evidence indicated that PEI fragments or significant amounts of amines were on the CDs’ surfaces. An attractive feature of the PEI-CD-Dox particles was the facile coupling Dox to the particles with a high loading capacity of 35.9%. Notably, the PEI-CD-Dox inhibited liver tumor cells (MHCC-97L and Hep3B) *in vitro* significantly more than free Dox. In contrast, free Dox was markedly more cytotoxic to normal liver cells than the PEI-CD-Dox treatment. *In vivo*, there was nearly complete inhibition of hepatocellular carcinoma growth in a mouse model [52]. Tumors treated with the Dox-loaded CDs were 50 mm^3^, while tumors treated with free Dox or phosphate buffer saline (PBS) were about 400 and 600 mm^3^, respectively. Consistent with these inhibitory results, the PEI-CD-Dox treatment accumulated more than the free Dox in the tumor xenografts. Moreover, the pH dependency and release of Dox of 70% at pH 5.2 correlated with the *in vitro* and *in vivo* inhibitory studies of these CDs. Not all studies showed this correlation. For example, the previously discussed polymeric NP that released 90% of Dox within 12 hours had no greater cytotoxicity than the pH-independent NPs [44].

**Fig. 5.**
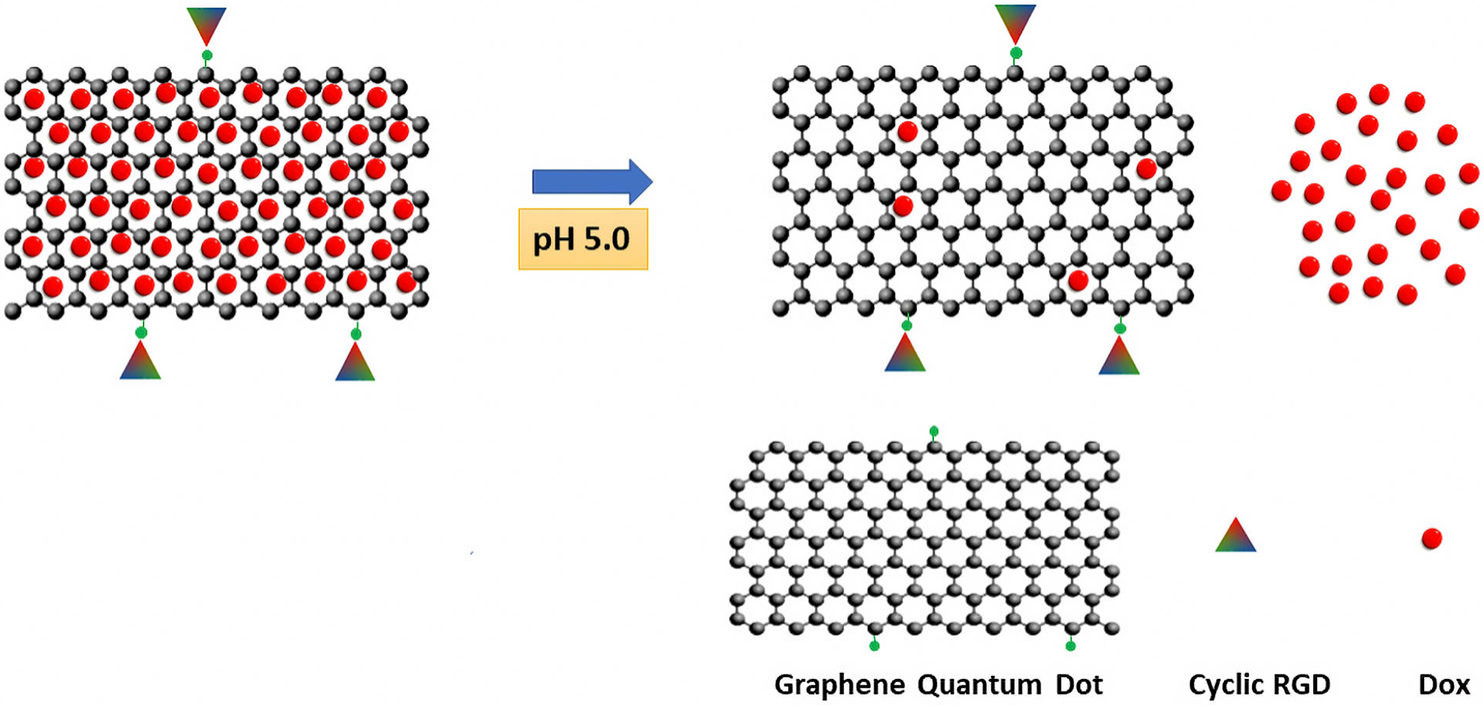
Flat honeycomb quantum carbon dot binds Dox efficiently at neutral pH and releases Dox at acidic pH. The cRGD ligand was conjugated to carboxyl groups on the surface of the graphene quantum dots (GQDs). The figure was created using Inkscape Version 1.2 (Inkscape Project, Boston, MA, USA).

Like carbon dots, graphene quantum dots (GQDs) are small, fluorescent, and biocompatible nanocarriers of drugs [47]. Unlike carbon dots, which form spheres, GQDs are flat (made up of 1 to 3 layers), and their basic building block is a honeycomb lattice. Both CDs and GQDs are part of a general class of carriers called carbon-based quantum dots. Interestingly, GQDs made from graphene oxide bind Dox efficiently at neutral pH and release the Dox at acidic pH [27]. The release of Dox from the GQDs occurred without any further modification of the surface of these GQDs (Fig. 5). The investigators thought that the pH dependency of these GQDs for Dox was based on reduced ionic interactions and hydrogen bonding (i.e., increased protonation of carboxyl groups on GQD’s surface) at acidic pH and the increased solubility of Dox (pH, 5.0). Moreover, surface functionalization of the Dox-loaded GQDs with the cyclic RGD ligand resulted in greater cytotoxicity to the cancer cells than those without the ligand [27].

Both drug-free CDs and GQDs carriers showed little to no toxicity on several cell lines [27,52]. Moreover, indicative of low toxicity, the weight of mice treated with CD was more than those treated with free Dox [52]. Other *in vitro* and *in vivo* studies have suggested the low toxicity of carbon dots [48]. Of some concern for their long-term treatment of human cancers is the reduced biodegradability of these chemical-inert carriers, resulting in their accumulation in tissues [55-57]. On the other hand, the parent carbon-based quantum dots (CDs and GQDs) are small (<10 nm) and should be excreted by the kidneys [58].

## Discussion and Future Directions.

Except for the NRP-1 targeted tetrahedron structure, the remainder of the drug-loaded particles would likely accumulate in the tumor *in vivo* by the EPR effect [59]. The EPR effect is due to a combination of leakiness of tumor blood vessels resulting in flux of NPs from the blood into the tumor tissue and reduced numbers of lymphatic vessels in tumors. Because tumor vessel fenestrations are between 100 and 300 nm [60], larger particles would likely not be effective in tumor inhibition *in vivo*. Indeed, of the drug-loaded particles between 30 and 100 nm, the smallest size particles had the most antitumor activity in an animal model [61]. Thus, the PMSA-targeted rolling circle and the folateligand PEI cyclodextrin carriers with a size of 234 nm and 367 nm, respectively, probably will have decreased tumor accumulation and antitumor activity [25,39].

Alternatively, targeting the neuropilin-1 (NRP-1) transport system, which augments transcytosis across the tumor endothelium, may enable larger particles to accumulate in the tumor and circumvent the size restrictions of EPR [62,63]. By replacing the PMSA ligand with an NRP-1 ligand in the rolling circle, the size of the rolling circle should be less limiting in the particles’ tumor accumulation [62]. In addition to large particles, targeting the NRP-1 pathway can also increase the transport of smaller particles (i.e., 60 nm NRP-1 targeted tetrahedron) into tumors, as observed with similarly sized NRP-1 targeted particles [63].

At the other end of the size spectrum, particles less than 8–10 nm will probably be secreted by the kidney [64,65]. Rapid secretion of these particles by the kidney may limit their accumulation in the tumor and antitumor efficacy. In this report, the size of the Dox-free parent CD was less than 10 nm, but the size of the Dox-loaded CD was not given [52]. Although marked tumor accumulation did occur with the Dox-loaded CD suggesting a size greater than 10 nm, the small size of the modified CDs and QCDs, in general, deserves scrutiny to ensure the size is above 10 nm to enhance antitumor efficacy.

Several other areas for these pH-dependent drug-loaded carriers will require more investigation, including *in vivo* stability and antitumor efficacy. Of the drug-loaded particles discussed, only two were investigated for their ability to inhibit tumors in animal models [23,52]. Furthermore, one of these examined *in vivo* biodistribution of the particles in the tumor and normal tissues [23,52]. Neither *in vivo* study determined the pharmacokinetics of the particles. Notably, a minimal half-life of 6 hours has been suggested to maximize the tumor accumulation of drug-loaded particles by EPR [66]. *In vitro* stability of these drug-loaded particles will also need further study. Although some non-covalent Dox-loaded particles demonstrated marked drug release at acidic pHs with minimal release at neutral pH, there were no stability studies beyond three days for any Dox-loaded particles in this review [52]. Regardless of their stability in aqueous solutions, these drug-loaded carriers will likely require lyophilization for clinical studies.

## Conclusion

We have reported on several pH-sensitive non-covalent interactions between carriers and chemotherapy agents in this review. Hydrogen and ionic bonds were primarily responsible for these interactions between drugs (Dox, *β*-lapachone, and Mtx) and diverse carriers, such as DNA, cyclodextrin, polymeric nanoparticles, and carbon dots. Furthermore, most of the pH-dependent drug-loaded particles showed increased cytotoxicity toward malignant cells. Moreover, the two drug (Dox)-loaded particles, a DNA micelle and carbon dot, that were investigated *in vivo*, demonstrated marked antitumor activity. Despite their promise, the pH-dependent drug-loaded particles are in their early stages of development and will require further investigations to determine their clinical applicability.
